# Experience-based integral reinforcement learning consensus for unknown multi-agent systems

**DOI:** 10.1038/s41598-025-15573-w

**Published:** 2025-09-26

**Authors:** Longquan Ma, Huarong Zhao, Yuhao Chen, Yi Gao, Hongnian Yu

**Affiliations:** 1 Engineering Research Center of Internet of Things Applications Ministry of Education, Jiangnan University, Wuxi, 214122 Jiangsu China; 2 Jiangsu Provincial Sensor Network Engineering Technology Research Center, Wuxi Institute of Technology, Wuxi, 214121 Jiangsu China; 3 School of Computing, Engineering and the Built Environment, Edinburgh Napier University, EH10 5DT Edinburgh, UK

**Keywords:** Integral reinforcement learning, Multi-agent systems, Consensus control, Energy harvesting, Energy storage, Renewable energy, Applied mathematics, Information technology, Applied physics, Electronics, photonics and device physics, Information theory and computation, Statistical physics, thermodynamics and nonlinear dynamics

## Abstract

This paper investigates an optimal consensus control problem and proposes a policy iteration algorithm based on online integral reinforcement learning for nonlinear multi-agent systems with unknown dynamics. Introducing a critic-actor neural network into the traditional policy iteration avoids the identification of unknown dynamics. To address the issue of local optima in online learning, an experience-based weight-tuning law is introduced to ensure the persistence of excitation conditions during the training phase. The theoretical results show that the system is asymptotically stable, and the network weights converge. Finally, the effectiveness and correctness are verified by several simulation studies.

## Introduction

The consensus optimal control problem of multi-agent systems (MASs) has been a highly active research area due to the multitude of applications in different areas, such as satellite scheduling^1^, wireless sensor networks^2^, multi-quadrotor formation flight^3^, robotics^4^, and vehicle formation control^5^. The primary objective of optimal control for MASs is to stabilize the system with the minimum tracking error and the energy consumed by a designed optimal controller^6^. Typically, the optimal controller is derived from the Hamilton-Jacobi-Bellman (HJB) equation, which is formulated based on a predefined performance index function^7^. However, solving the HJB equation is challenging due to its inherent nonlinearity and partial derivatives, making an analytical solution practically infeasible^8,9^. As an alternative, the policy iteration (PI) algorithm has been widely adopted as an effective method for approximating the solution to the HJB equation^10^. Instead of solving the equation directly, PI iteratively alternates between policy evaluation and improvement until convergence is achieved^11^. Nevertheless, most PI algorithms^12–14^ require that the dynamics of the controlled MASs are known. It should be pointed out that accurate dynamics of MASs are hard to obtain because of complex constructions and aging of components^15,16^. Therefore, developing a PI scheme that operates independently of system dynamics is a critical and meaningful research direction.

As the issue mentioned above, it is noted that neural networks are often introduced to design a controller for the controlled system with unknown or uncertain dynamics^17–20^. For example, Ferede et al.^21^ designed an end-to-end neural network controller for an aggressive high-speed quadcopter, Zishan et al.^22^ formulated an implementation of a densely connected neural network to detect arrhythmia on a low-compute device, and Hu et al.^23^ investigated a neural network-based robust tracking control algorithm for multi-motor driving servo systems. While these approaches effectively reduce control errors for systems with partially unknown dynamics, they often overlook the energy consumption of the designed controllers^21–23^. Motivated by this limitation, this paper aims to integrate a neural network-based approach into the PI framework to achieve optimal control while considering energy efficiency.

Recently, Modares et al.^24^ studied an integral reinforcement learning (IRL) method to address the challenges above, constructing an actor-critic neural network framework based on the PI algorithm. Building on this foundation, several advanced IRL approaches have been proposed. For example, Shen et al.^25^ studied an IRL method for nonlinear Markov jump singularly perturbed systems, Lin et al.^26^ designed a dynamic compensator-based IRL approach for unknown nonaffine nonlinear systems, and Yan et al.^27^ investigated a graphical game-based IRL bipartite containment scheme for high-order nonlinear MASs. However, most existing IRL methods^28–31^ face the risk of being trapped in local optima. While exploration and target strategies^32–34^ have been proposed to mitigate this issue, they are primarily suited for offline learning, which demands significant computational and storage resources. Consequently, a key motivation of this paper is to develop an online learning IRL approach that avoids local optima while maintaining computational efficiency by utilizing historical information over a specific period.

This paper investigates an optimal consensus control problem for MASs with unknown dynamics and proposes a PI algorithm based on online integral reinforcement learning with experience data. The main contributions of this article are listed as follows:

1. An actor-critic neural network-based PI algorithm is designed for nonlinear MASs. Unlike existing methods^35,36^, the proposed approach does not require prior knowledge of system dynamics or relies on a neural network-based identifier to approximate unknown dynamics, thereby avoiding additional cumulative errors.
2. An experience-based IRL method is formulated that bridges online and offline learning schemes for nonlinear MASs. Compared with existing methods^37,38^, the proposed approach selectively utilizes historical information over a specific period, effectively preventing convergence to local optima.

*Notations*: $R^n$ and $R^{n \times m}$ denote the set of *n*-dimensional real vectors and $n \times m$ real matrices, respectively. $\underline{1} \in R^n$ denotes the *n*-dimensional vector, where all elements are 1. ||*a*|| denotes the Euclidean norm of $a \in R^n$, ${I_n}$ denotes an identity matrix with *n* dimensions, and $\otimes$ denotes the Kronecker product.

## Preliminaries and problem formulations

Here, we introduce fundamental knowledge and notations used in this article and describe the consensus issue of MASs.

### Graph theory

Define a directed graph $\mathscr {G} = \left( {\mathscr {V},\mathscr {E},\mathscr {A}} \right)$, which is composed of a finite nonempty node set $\mathscr {V} = \{ 1,2,...,N\}$, an edge set $\mathscr {E} \subseteq \mathscr {V} \times \mathscr {V}$, and a weighted adjacent matrix $\mathscr {A} = \left[ {{a_{ij}}} \right] \in {\mathscr {R}^{N \times N}}$. If node *j* can send information to node *i*, it has $(j,i) \in \mathscr {E}$, ${a_{ij}}> 0$ (otherwise ${a_{ij}} = 0$), and node *j* is one of the neighbors of node *i*. Define ${\mathscr {N}_i} = \left\{ {{j}:\left( {{j},{i}} \right) \in \mathscr {E}} \right\}$ as the set of all the neighbors of node *i*. Define the in-degree matrix $\mathscr {D}\, = diag\left( {{d_1},...,{d_N}} \right)$, where ${d_i} = \sum \nolimits _{j \in {\mathscr {N}_i}} {{a_{ij}}}$, and the Laplacian matrix $\mathscr {L}$ is defined as $\mathscr {L} = \mathscr {D} - \mathscr {A} = [l_{ij}]$.

### Consensus of MASs

Consider MASs in the form of a communication network ${\mathscr {G}}$ consisting of *N* agents, where the dynamics of agent *i* is given as

1 $$\begin{aligned} {\dot{x}_i} = f\left( {{x_i}} \right) + g\left( {{x_i}} \right) {u_i},i = 1,...,N \end{aligned}$$

where $f\left( {{x_i}} \right) \in {R^n}$ are partially unknown nonlinear smoothly function, and $g\left( {{x_i}} \right) \in {R^{n \times m}}$ is partially unknown control effectiveness function with $||g\left( {{x_i}} \right) ||<\bar{g}$. Moreover, $f\left( {{x_i}} \right) \in {R^n}$ and $g\left( {{x_i}} \right)$ are Lipschitz continuous. ${x_i} = {x_i}\left( t \right)$ is the state vector, and ${u_i} = {u_i}\left( t \right) \in {R^m}$ is the control input vector of agent *i*.

The global network dynamics is

2 $$\begin{aligned} \dot{x} = f\left( x \right) + g\left( x \right) u \end{aligned}$$

where $x = {\left[ {x_1^ \top , \hspace{5.0pt}x_2^ \top , \hspace{5.0pt}..., \hspace{5.0pt}x_N^ \top } \right] ^ \top } \in {R^{Nn}}$, $f\left( x \right) = {\left[ {{f^ \top }\left( {{x_1}} \right) ,\hspace{5.0pt}{f^ \top }\left( {{x_2}} \right) ,\hspace{5.0pt}...,\hspace{5.0pt}{f^ \top }\left( {{x_N}} \right) } \right] ^ \top } \in {R^{Nn}}$, $g\left( x \right) = diag\left( {g\left( {{x_i}} \right) } \right) \in {R^{Nn \times Nm}}$ with $i = 1,2,...,N$, and $u = {\left[ {u_1^ \top \hspace{5.0pt}u_2^ \top \hspace{5.0pt}...\hspace{5.0pt}u_N^ \top } \right] ^ \top } \in {R^{Nm}}$.

#### Assumption 1

^39^The system (2) is controllable on a set $\Omega \in {R^{Nn}}$, which implies that there exists a control policy that can asymptotically stabilize the system.

#### Assumption 2

^40^A directed spanning tree is present in the communication network ${\mathscr {G}}$, and all agents have direct or indirect access to the leader agent’s information.

The leader state vector ${x_0} = {x_0}\left( t \right) \in {R^n}$ satisfies that

3 $$\begin{aligned} {\dot{x}_0} = k\left( {{x_0}} \right) \end{aligned}$$

where $k\left( {{x_0}} \right) \in {R^n}$. The tracking error of agent *i* is defined as

4 $$\begin{aligned} {\delta _i} = \sum \limits _{j \in {\mathscr {N}_i}} {{a_{ij}}\left( {{x_i} - {x_j}} \right) + {b_i}\left( {{x_i} - {x_0}} \right) } \end{aligned}$$

where ${\delta _i} = {\left[ {{\delta _{i1}}\hspace{5.0pt}{\delta _{i2}}\hspace{5.0pt}...\hspace{5.0pt}{\delta _{in}}} \right] ^ \top } \in {R^n}$. Note that ${b_i}> 0$ if and only if the leader agent can send information to agent *i*; otherwise, ${b_i} = 0$. The global error vector is given as

5 $$\begin{aligned} \delta = \mathscr {L}\left( {x - \underline{x} _0} \right) \end{aligned}$$

where $\mathscr {L} = \left( {L + B} \right) \otimes {I_n}$, $\delta = {\left[ {\delta _1^ \top \hspace{5.0pt}\delta _2^ \top \hspace{5.0pt}...\hspace{5.0pt}\delta _N^ \top } \right] ^ \top } \in {R^{Nn}}$ with $\underline{I} = \underline{1} \otimes {I_n} \in {R^{Nn \times n}}$, and $B = diag\left\{ {{b_1},{b_2},...,{b_N}} \right\} \in {R^{N \times N}}$(${b_{ii}} = {b_i}$ and ${b_{ij}} = 0,i \ne j$).

By differentiating Eq. (4), the dynamics of ${\delta _i}$ is

6 $$\begin{aligned} {{\dot{\delta }} _i} =&\; \left( {{L_i} + {B_i}} \right) \otimes {I_n}\left( \dot{x} - \dot{\underline{x}}_0 \right) \nonumber \\ =&\; \left( {{L_i} + {B_i}} \right) \otimes {I_n} \left( f\left( x \right) + g\left( x \right) u - k(\underline{x}_0) \right) \nonumber \\ =&\; \left( {{L_i} + {B_i}} \right) \otimes {I_n} \left( f_e\left( x \right) + g\left( x \right) u \right) \nonumber \\ =&\; \sum \limits _{j \in \{\mathscr {N}_i,i\}}\left( \left( l_{ij}+b_{ij} \right) \otimes {I_n}\right) \left( f(x_j)-k(x_0)+g(x_j)u_j\right) \nonumber \\ =&\; {\mathscr {L}_i}{f_e}\left( x \right) + \left( {{d_i} + {b_i}} \right) g\left( {{x_i}} \right) {u_i} - \sum \limits _{j \in {\mathscr {N}_i}} {{a_{ij}}g\left( {{x_j}} \right) {u_j}} \end{aligned}$$

where ${\mathscr {L}_i} = \left( {{L_i} + {B_i}} \right) \otimes {I_n}$. ${L_i} = \left[ {{l_{i1}}\hspace{5.0pt}...\hspace{5.0pt}{l_{ii}}\hspace{5.0pt}...\hspace{5.0pt}{l_{iN}}} \right]$ and ${B_i} = \left[ {{b_{i1}}\hspace{5.0pt}...\hspace{5.0pt}{b_{ii}}\hspace{5.0pt}...\hspace{5.0pt}{b_{iN}}} \right]$ are denoted as the *i*th row vector of *L* and *B*, respectively. Moreover, ${f_e}\left( x \right) = f\left( x \right) - \underline{k} \left( {{x_0}} \right)$ with $\underline{k} \left( {{x_0}} \right) = \underline{I} k\left( {{x_0}} \right)$.

### Problem statements

This paper aims to address the following problems and challenges.

1. How to solve the HJB equation for nonlinear MASs. To address this issue, we propose a PI scheme that is independent of system dynamics.
2. How to design a controller for MASs with unknown dynamics. To address this problem, we establish an actor-critic neutral network-based PI algorithm, where the dynamics are no longer needed.
3. How to avoid the designed IRL method from getting trapped in local optima. To address this drawback, we formulate an experience-based IRL method that selectively utilizes historical information to prevent getting trapped in local optima.

To sum up, the object of this study is to design a control strategy that ensures the uniform ultimate boundedness (UUB) of the system error ${\delta _i}$ for all $i \in \left\{ 0,1,..., N \right\}$ without relying on the system model. Specifically, there exist positive constants $\epsilon$ and $\bar{t}$ such that, for all initial conditions and under Assumption 1, the tracking error ${\delta _i}$ satisfies the following condition:

$$||\delta _i(t)||\le \epsilon , \forall t>\bar{t}$$

In other words, ${\delta _i}$ will enter and remain within a bounded region after time $\bar{t}$, thereby achieving the consensus control.

##  Controller design and convergence analysis

### Discont fator-based optimal control policy

Here, a distributed performance function with a discount factor is proposed to guarantee that each agent minimizes their performance function.

Define the discounted local performance index function of agent *i* as

7 $$\begin{aligned} {J_i}\left( {{\delta _i}\left( 0 \right) ,{u_i},{u_{ - i}}} \right) = \int _0^\infty {{e^{ - \alpha v}}r\left( {{\delta _i}\left( v \right) ,{u_i}\left( v \right) ,{u_{ - i}}\left( v \right) } \right) dv} \end{aligned}$$

where $r\left( {{\delta _i}\left( v \right) ,{u_i}\left( v \right) ,{u_{ - i}}\left( v \right) } \right) = {r_1}\left( {{\delta _i}} \right) + {r_2}\left( {{u_i},{u_{ - i}}} \right)$ denotes the cost function. ${r_1}\left( {{\delta _i}} \right) = \delta _i^ \top {Q_{ii}}{\delta _i}$ denotes the error cost, and ${r_2}\left( {{u_i},{u_{ - i}}} \right) = u_i^ \top {R_{ii}}{u_i} + \sum \nolimits _{j \in {\mathscr {N}_i}} {u_j^ \top {R_{ij}}{u_j}}$ denotes the control cost. $\alpha> 0$ is the discount factor. ${Q_{ii}} \geqslant 0$, ${R_{ii}}> 0$, and ${R_{ij}}> 0$ are positive symmetric matrices. For brief, $\sum \nolimits _{j \in {\mathscr {N}_i}} {u_j}$ is abbreviated to ${u_{ - i}}$.

We need to design a distributed optimal consensus method for each agent to ensure that all agents reach a consensus with the leader and reduce the local power function. In other words, this paper aims to minimize each agent’s local performance function with a designed control input set $\{ u_1, u_2,..., u_N \}$.

#### Definition 1

(Global Nash Equilibrium) For all $i = 1,2,...,N$, a set of control inputs $\{u_1^ *,u_2^ *,...,u_N^ * \}$ is considered to establish a global Nash equilibrium if the following equation is satisfied:

8 $$\begin{aligned} J_i^* = {J_i}\left( {{\delta _i}\left( {{t_0}} \right) ,u_i^*,u_{-i}^*} \right) \leqslant {J_i}\left( {{\delta _i}\left( {{t_0}} \right) ,u_i,u_{-i}^*} \right) ,\left( u_i \ne u_i^* \right) \end{aligned}$$

The local discounted value function of each agent *i* is defined as

9 $$\begin{aligned} {V_i}\left( {{\delta _i}\left( t \right) } \right) = \int _t^\infty {{e^{ - \alpha \left( {v - t} \right) }}r\left( {{\delta _i}\left( v \right) ,{u_i}\left( v \right) ,{u_{ - i}}\left( v \right) } \right) dv} \end{aligned}$$

The optimal local discounted value function is defined as

10 $$\begin{aligned} V_i^ * \left( {{\delta _i}\left( t \right) } \right) = \mathop {\min }\limits _{{u_1},{u_2},...,{u_N}} \int _t^\infty {{e^{ - \alpha \left( {v - t} \right) }}r\left( {{\delta _i}\left( v \right) ,{u_i}\left( v \right) ,{u_{ - i}}\left( v \right) } \right) dv} \end{aligned}$$

The local coupled Hamiltonian function is constructed as

11 $$\begin{aligned} {H_i}\left( {{\delta _i},\nabla {V_i},{u_i},{u_{ - i}}} \right)&= \delta _i^ \top {Q_{ii}}{\delta _i} + u_i^ \top {R_{ii}}{u_i} + \sum \limits _{j \in \mathscr {N}_i} {u_j^ \top {R_{ij}}{u_j}} \nonumber \\&\quad \; + \nabla V_i^ \top \left( {{\mathscr {L}_i}{f_e}\left( x \right) + \left( {{d_i} + {b_i}} \right) g\left( {{x_i}} \right) {u_i} - \sum \limits _{j \in {\mathscr {N}_i}} {{a_{ij}}g\left( {{x_j}} \right) {u_j}} } \right) \end{aligned}$$

The gradient of the value function ${V_i}$ with respect to ${\delta _i}\left( t \right)$ is denoted by $\nabla {V_i}$. $V_i^ * \left( {{\delta _i}} \right)$ denotes the local optimal value function that satisfies

12 $$\begin{aligned} \mathop {\min }\limits _{{u_i}} {H_i}\left( {{\delta _i},\nabla V_i^ *,{u_i},{u_{ - i}}} \right) = 0 \end{aligned}$$

The optimal control policy can minimize Eq. (12), which can be expressed as

13 $$\begin{aligned} \frac{{\partial {H_i}}}{{\partial {u_i}}} = 0 \rightarrow u_i^ * = - \frac{1}{2}\left( {{d_i} + {b_i}} \right) R_{ii}^{ - 1}{g^ \top }\left( {{x_i}} \right) \nabla V_i^ * \end{aligned}$$

### An IRL-based PI algorithm

Substituting Eq. (13) into Eq. (12), we have

14 $$\begin{aligned} \delta _i^ \top {Q_{ii}}\delta&+ \frac{1}{4}{\left( {{d_i} + {b_i}} \right) ^2}{\left( {\nabla V_i^ * } \right) ^ \top }g\left( {{x_i}} \right) R_{ii}^{ - 1}g{\left( {{x_i}} \right) ^ \top }\nabla V_i^ * \nonumber \\&+ \frac{1}{4}\sum \limits _{j \in {\mathscr {N}_i}} {{{\left( {{d_j} + {b_j}} \right) }^2}{{\left( {\nabla V_j^ * } \right) }^ \top }g\left( {{x_j}} \right) R_{jj}^{ - 1}{R_{ij}}R_{jj}^{ - 1}g{{\left( {{x_j}} \right) }^ \top }\nabla V_j^ * } \nonumber \\&+ {\left( {\nabla V_i^ * } \right) ^ \top }\left( {{\mathscr {L}_i}{f_e}\left( x \right) - \frac{1}{2}{{\left( {{d_i} + {b_i}} \right) }^2}g\left( {{x_i}} \right) R_{ii}^{ - 1}g{{\left( {{x_i}} \right) }^ \top }\nabla V_i^ * } \right) \nonumber \\&+ {\left( {\nabla V_i^ * } \right) ^ \top }\left( {\frac{1}{2}\sum \limits _{j \in {\mathscr {N}_i}} {{a_{ij}}\left( {{d_j} + {b_j}} \right) g\left( {{x_i}} \right) R_{jj}^{ - 1}g{{\left( {{x_i}} \right) }^ \top }\nabla V_j^ * } } \right) = 0 \end{aligned}$$

It is noted that in solving Eq. (14), the optimal control policy of the agents is obtained. However, since Eq. (14) contains the information of the system’s dynamics, which is unknown, the solution of Eq. (14) is difficult to obtain. A common approach to solving this problem is using the PI algorithm, which approximates the solution through constant iteration. The PI algorithm consists of two steps: 1) Policy evaluation and 2) Policy improvement. In the policy evaluation step, according to Eq. (11) and Eq. (12), the given control policy $u_i^{(k)}(t)$ is evaluated by

15 $$\begin{aligned} 0&= {r_i}\left( {{\delta _i},u_i^{\left( k \right) },u_j^{\left( k \right) }} \right) + {\left( {\nabla V_i^{\left( k \right) }} \right) ^ \top }\nonumber \\&\quad \;\times \left[ {{{{\mathscr {L}}}_i}{f_e}\left( x \right) + \left( {{d_i} + {b_i}} \right) g\left( {{x_i}} \right) {u^{\left( k \right) }} - \sum \limits _{j \in {{{\mathscr {N}}}_i}} {{a_{ij}}g\left( {{x_j}} \right) u_j^{\left( k \right) }} } \right] \end{aligned}$$

and updates it by

16 $$\begin{aligned} u_i^{\left( {k + 1} \right) }\left( t \right) = - \frac{1}{2}\left( d_i+b_i\right) R_{ii}^{ - 1}{g^ \top }\left( {{x_i}} \right) \nabla V_i^{\left( k \right) } \end{aligned}$$

From Eqs. (15) and (16), it is found that they incorporate the dynamics of the controlled system. In order to release the need for the system’s dynamics, an IRL algorithm with an integration interval *T* is introduced into Eq. (9) that yields

17 $$\begin{aligned} {V_i}\left( {{\delta _i}\left( t \right) } \right) = \int _{t}^{t+T} {{e^{ - \alpha \left( {v - t} \right) }}r\left( {{\delta _i}\left( v \right) ,{u_i}\left( v \right) ,{u_{ - i}}\left( v \right) } \right) dv} + e^{-\alpha T}{V_i}\left( {{\delta _i}\left( t+T \right) } \right) \end{aligned}$$

where the controlled system’s dynamics are not contained.

Then, an IRL-based PI algorithm is designed as Algorithm 1.

**Algorithm 1:**
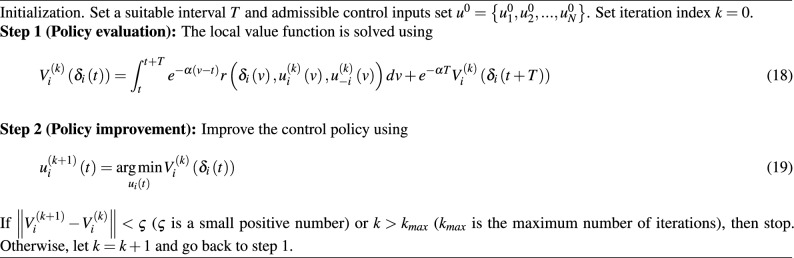
IRL-Based Policy Iteration

Due to Algorithm 1 including an integral interval *T*, the update of control inputs is discontinuous. Thus, the designed IRL-based PI scheme can be regarded as a time-triggered control strategy, where *T* represents a special sampling period.

#### Remark 1

Compared with the common PI, the designed IRL-based PI eliminates the requirements for system dynamics. Moreover, according to the analysis in the work of Lewis et al.^42^, it is obtained that Eqs. (15) and (18) are equivalent. However, it also introduces a new problem: How to solve Eq. (19), presented below.

### Model-free distributed consensus control algorithm

Here, we constructed a critic neural network and an actor neural network for Eqs. (18) and (19), respectively. Moreover, the weights tuning laws are designed, and the convergence is analyzed.

According to the universal approximation theorem^43^, it is obtained that neural networks can approximate smooth functions on a compact set. Thus, we construct neural networks to estimate the objective function, where the established neural networks consist of three layers: 1) the input layer, 2) the hidden layer, and 3) the output layer. The input-to-hidden weight is set to be 1 and no longer tuned. The hidden layer contains an activation function, and the weight connecting the hidden layer to the output can be tuned to minimize the approximation error. Eqs. (18) and (19) is expressed as

20 $$\begin{aligned} & {\hat{V}}_i^{(k)}\left( {\delta _i}\left( t \right) \right) = {\left( {{\hat{W}}_{ci}^{(k),(p)}} \right) ^ \top }{\phi _{ci}}\left( {{z_{ci}}\left( t \right) } \right) \end{aligned}$$

21 $$\begin{aligned} & {\hat{u}}_i^{(k)}\left( t \right) = {\left( {{\hat{W}}_{ai}^{(k),(p)}} \right) ^ \top }{\psi _{ai}}\left( {{z_{ai}}\left( t \right) } \right) \ \end{aligned}$$

where ${\phi _{ci}}\left( {{z_{ci}}\left( t \right) } \right) \in {R^{{h_{vi}}}}$, and ${\psi _{ai}}\left( {{z_{ai}}\left( t \right) } \right) \in {R^{{h_{di}}}}$ denote the activation function vectors. ${h_{vi}}$ and ${h_{di}}$ are the numbers of neurons. ${\hat{W}}_{ci}^k$ and ${\hat{W}}_{ai}^k$ denote the weight vectors. ${z_{ci}}\left( t \right)$ is a vector of the information from ${\delta _i}\left( t \right)$, $u_i^k\left( t \right)$, and $u_{ - i}^k\left( t \right)$. ${z_{ai}}\left( t \right)$ denotes a vector of the information from ${\delta _i}\left( t \right)$.

Based on Eq. (18), the approximation error of the critic neural network is defined as

22 $$\begin{aligned} e_{ci}^{(k)}\left( t \right)&= \int _t^{t + T} {{e^{ - \alpha \left( {v - t} \right) }}{r_i}\left( {{\delta _i}\left( v \right) ,u_i^{(k)}\left( v \right) ,u_{ - i}^{(k)}\left( v \right) } \right) } dv \nonumber \\&\quad \; + {e^{ - \alpha T}}{\hat{V}}_i^{(k)}\left( {t + T} \right) - {\hat{V}}_i^{(k)}\left( t \right) \end{aligned}$$

To make the square error $E_{ci}^{(k)}\left( t \right) = \left( {1/2} \right) {\left( {e_{ci}^{(k)}\left( t \right) } \right) ^ \top }e_{ci}^{(k)}\left( t \right)$ minimal, a gradient-based update rule with history data for the critic neural network weights of agent *i* is derived as

23 $$\begin{aligned} {\hat{W}}_{ci}^{(k + 1)} =&\; {\hat{W}}_{ci}^{(k)} - {\beta _{ci}}\int _{t - s}^t {\frac{{{e^{v - t + s}}}}{{\int _{t - s}^t {{e^{v - t + s}}dv} }}\left[ {\frac{{\partial E_{ci}^{(k)}\left( v \right) }}{{\partial {\hat{W}}_{ci}^{(k)}}}} \right] dv} \nonumber \\ =&\; {\hat{W}}_{ci}^{(k)} - {\beta _{ci}}\int _{t - s}^t {\frac{{{e^{v - t + s}}}}{{{e^s} - 1}}\frac{{\partial E_{ci}^{(k)}\left( v \right) }}{{\partial e_{ci}^{(k)}\left( v \right) }}\frac{{\partial e_{ci}^{(k)}\left( v \right) }}{{\partial {\hat{V}}_i^{(k)}\left( v \right) }}\frac{{\partial {\hat{V}}_i^{(k)}\left( v \right) }}{{\partial {\hat{W}}_{ci}^{(k)}}}dv} \nonumber \\ =&\; {\hat{W}}_{ci}^{(k)} - {\beta _{ci}}\int _{t - s}^t {\frac{{{e^{v - t + s}}}}{{{e^s} - 1}}{e^{ - \alpha T}}e_{ci}^{(k)}\left( v \right) {\phi _{ci}}\left( {{z_{ci}}\left( v \right) } \right) dv} \end{aligned}$$

where ${\beta _{ci}}> 0$ is the learning rate, and *s* denotes the time range of the history data, which could be used to update the weights.

The approximation error of the actor neural network is defined as

24 $$\begin{aligned} e_{ai}^{(k)}\left( t \right) = {\hat{V}}_i^{(k)}\left( t \right) \end{aligned}$$

Define the square error as $E_{ai}^{(k)} = \left( {1/2} \right) {\left( {e_{ai}^{(k)}} \right) ^ \top }e_{ai}^{(k)}$, and the update rule for the actor neural network weights of agent *i* is derived as

25 $$\begin{aligned} {\hat{W}}_{ai}^{(k + 1)}=&\; {\hat{W}}_{ai}^{(k)} - {\beta _{ai}}\int _{t - s}^t {\frac{{{e^{v - t + s}}}}{{\int _{t - s}^t {{e^{v - t + s}}dv} }}\left[ {\frac{{\partial E_{ai}^{(k)}\left( v \right) }}{{\partial {\hat{W}}_{ai}^{(k)}}}} \right] dv} \nonumber \\ =&\; {\hat{W}}_{ai}^{(k)} - {\beta _{ai}}\int _{t - s}^t {\frac{{{e^{v - t + s}}}}{{{e^s} - 1}}\frac{{\partial E_{ai}^{(k)}\left( v \right) }}{{\partial e_{ai}^{(k)}\left( v \right) }}\frac{{\partial e_{ai}^{(k)}\left( v \right) }}{{\partial {\hat{V}}_i^{(k)}\left( v \right) }}} \nonumber \\&\times \frac{{\partial {\hat{V}}_i^{(k)}\left( v \right) }}{{\partial {\phi _{ci}}\left( {{z_{ci}}\left( v \right) } \right) }}\frac{{\partial {\phi _{ci}}\left( {{z_{ci}}\left( v \right) } \right) }}{{\partial {z_{ci}}\left( v \right) }}\frac{{\partial {z_{ci}}\left( v \right) }}{{\partial {\hat{u}}_i^{(k)}\left( v \right) }}\frac{{\partial {\hat{u}}_i^{(k)}\left( v \right) }}{{\partial {\hat{W}}_{ai}^{(k)}}}dv \nonumber \\ =&\; {\hat{W}}_{ai}^{(k)} - {\beta _{ai}}\int _{t - s}^t {\frac{{{e^{v - t + s}}}}{{{e^s} - 1}}{\psi _{ai}}\left( {{z_{ai}}\left( v \right) } \right) {{\left( {{\hat{W}}_{ci}^{(k)}} \right) }^ \top }} \nonumber \\&\times {{\phi '}_{ci}}\left( {{z_{ci}}\left( v \right) } \right) {Z_i}{\left[ {{{\left( {{\hat{W}}_{ci}^{(k)}} \right) }^ \top }{\phi _{ci}}\left( {{z_{ci}}\left( v \right) } \right) } \right] ^ \top }dv \end{aligned}$$

where ${\beta _{ai}}> 0$ is the learning rate, ${Z_i} = \partial {z_{ci}}\left( t \right) /\partial {\hat{u}}_i^{(k)}\left( t \right)$, and ${\phi '_{ci}}\left( {{z_{ci}}\left( t \right) } \right) = \partial {\phi _{ci}}\left( {{z_{ci}}\left( t \right) } \right) /\partial {z_{ci}}\left( t \right)$.

Combining the critic-actor neural network framework with Algorithm 1 yields the following algorithm, as shown in Algorithm 2.

**Algorithm 2:**
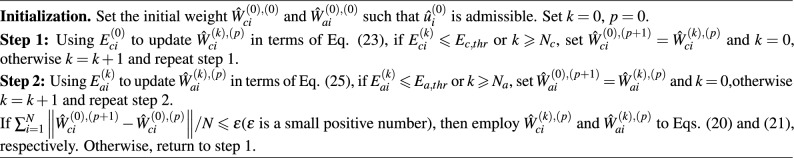
Model-free Distributed Consensus Control Algorithm.

#### Remark 2

Notably, this weight update rule utilizes historical data, and a replay buffer is established to store this data, similar to references^37,38^. However, those update rules do not assign ratios to different periods of historical data. This paper introduces the exponential term for importance sampling, which allows the proportion of history data in the weight update to be inversely proportional to the time interval $t-s^{\prime }$ between the historical moment $s^{\prime }$ $(t-s<s^{\prime }<t)$ and the current moment *t*. The reason for importance sampling is that control inputs and states closer to the current moment have a more substantial influence on the state at the current moment. This weight update rule prevents the critic neural network from making inaccurate approximations of the past states of the agents, improves the control policy estimated by the actor neural network for real-time control processes, and accelerates the convergence of weights.

### Convergence analysis

#### Theorem 1

Let the update rules for critic and actor neutral network weights be as in Eqs. (23) and (25). Define the weights estimation errors ${\tilde{W}}_{ci}^{(k)} = {\hat{W}}_{ci}^{(k)} - W_{ci}^ *$ and ${\tilde{W}}_{ai}^{(k)} = {\hat{W}}_{ai}^{(k)} - W_{ai}^ *$. Then, ${\tilde{W}}_{ci}^{(k)}$, ${\tilde{W}}_{ai}^{(k)}$, and $\delta _i$ are UUB as ${k} \rightarrow \infty$, and there exists a scalar $W> 0$, which satisfies

26 $$\begin{aligned} 2{\beta _i^ \top }\int _{t - s}^t {{\rho _i}\left( v \right) \eta _i \left( v \right) dv}>&{\left\| {\int _{t - s}^t {\beta _i^{\top } {\rho _i ^{}}\left( v \right) \lambda _i \left( v \right) dv} } \right\| ^2} + \beta _i^\top \int _{t - s}^t {{\rho _i }\left( v \right) \lambda _i \left( v \right) dv}\underline{W} \end{aligned}$$

#### Proof

By Eqs. (23) and (25), ${\tilde{W}}_{ci}^{(k)}$ and ${\tilde{W}}_{ai}^{(k)}$ are rewritten as

27 $$\begin{aligned} & {\tilde{W}}_{ci}^{(k + 1)} = {\tilde{W}}_{ci}^{(k)} - {\beta _{ci}}\int _{t - s}^t {{\rho _{ci}}\left( v \right) e_{ci}^{(k)}\left( v \right) dv} \end{aligned}$$

28 $$\begin{aligned} & {\tilde{W}}_{ai}^{(k + 1)} = {\tilde{W}}_{ai}^{(k)} - {\beta _{ai}}\int _{t - s}^t {{\rho _{ai}}\left( v \right) {\hat{V}}_i^{(k)}\left( v \right) dv} \end{aligned}$$

where

$$\begin{aligned} & {\rho _{ci}}\left( v \right) = \frac{e^{v - t + s}}{e^s - 1} {e^{ - \alpha T}}{\phi _{ci}}\left( {{z_{ci}}\left( v \right) } \right) \\ & {\rho _{ai}}\left( v \right) = \frac{e^{v - t + s}}{e^s - 1} {\psi _{ai}}\left( {{z_{ai}}\left( v \right) } \right) {\left( {{\hat{W}}_{ci}^{(k)}} \right) ^ \top }{\phi '_{ci}}\left( {{z_{ci}}\left( v \right) } \right) {Z_i} \end{aligned}$$

Choose a Lyapunov function as

29 $$\begin{aligned} {L_i}\left( k \right) = {L_{i,1}}\left( k \right) + {L_{i,2}}\left( k \right) +{L_{i,3}}\left( k \right) \end{aligned}$$

where $${L_{i,1}}\left( k \right) = {\left( {{\tilde{W}}_{ci}^{(k)}} \right) ^ \top }{\tilde{W}}_{ci}^{(k)}$$, $${L_{i,2}}\left( k \right) = {\left( {{\tilde{W}}_{ai}^{(k)}} \right) ^ \top }{\tilde{W}}_{ai}^{(k)}$$, and $${L_{i,3}}\left( k \right) = \delta _i^\top \delta _i + \Theta _iV_i(\delta _i(t))$$.

The gradient of ${L_{i,1}}\left( k \right)$ is obtained as

30 $$\begin{aligned} \nabla {L_{i,1}}\left( k \right) =&{\left( {{\tilde{W}}_{ci}^{(k + 1)}} \right) ^\top }{\tilde{W}}_{ci}^{(k + 1)} - {\left( {{\tilde{W}}_{ci}^{(k)}} \right) ^\top }{\tilde{W}}_{ci}^{(k)} \nonumber \\ =&- 2{\beta _{ci}}\int _{t - s}^t { \left( {\rho _{ci}}\left( v \right) e_{ci}^{(k)}\left( v \right) \right) ^\top dv} \left( {{\hat{W}}_{ci}^{(k)} - {W_{ci}^{(k)}} + {W_{ci}^{(k)}} - W_{ci}^ * } \right) + \beta _{ci}^2{\left\| {\int _{t - s}^t {{\rho _{ci}}\left( v \right) e_{ci}^{(k)}\left( v \right) dv} } \right\| ^2} \end{aligned}$$

where $${W_{ci}^{(k)}}$$ satisfies

31 $$\begin{aligned} \left( {{\phi _{ci}}\left( {{z_{ci}}\left( t \right) } \right) - {e^{ - \alpha T}}{\phi _{ci}}\left( {{z_{ci}}\left( {t + T} \right) } \right) } \right) ^\top W_{ci}^{(k)} = \int _t^{t + T} {{e^{ - \alpha \left( {v - t} \right) }}{r_i}\left( {{\delta _i}\left( v \right) ,u_i^{(k)}\left( v \right) ,u_{ - i}^{(k)}\left( v \right) } \right) } dv \end{aligned}$$

Because $W_{ci}^{(k)}$ is bounded, there exists a scalar ${W_1}> 0$ which satisfies $\left\| {W_{ci}^{(k)} - W_{ci}^ * } \right\| ^2 \leqslant {W_1}$. Substituting Eq. (31) into Eq. (22), one has

32 $$\begin{aligned} e_{ci}^{(k)}\left( t \right) = \left( {{e^{ - \alpha T}}{\phi _{ci}}\left( {{z_{ci}}\left( {t + T} \right) } \right) - {\phi _{ci}}\left( {{z_{ci}}\left( t \right) } \right) } \right) ^\top \left( {{\hat{W}}_{ci}^{(k)} - W_{ci}^{(k)}} \right) \end{aligned}$$

According to Eq. (32), Eq. (30) is rewritten as

33 $$\begin{aligned} \nabla {L_{i,1}}\left( k \right)&=\; {\beta _{ci}}\left( {{\beta _{ci}}{{\left\| {\int _{t - s}^t {{\rho _{ci}}\left( v \right) e_{ci}^{(k)}\left( v \right) dv} } \right\| }^2} - 2\int _{t - s}^t {{\rho ^\top _{ci}}\left( v \right) {\eta _{ci}}\left( v \right) dv} } \right) \nonumber \\&+ \beta _{ci}^{}\int _{t - s}^t {\left( {\rho _{ci}}\left( v \right) e_{ci}^{(k)}\left( v \right) \right) ^\top dv} \left( {W_{ci}^ * - W_{ci}^{(k)}} \right) \nonumber \\ \leqslant&\; {\beta _{ci}}\left( {{\beta _{ci}}{{\left\| {\int _{t - s}^t {{\rho _{ci}}\left( v \right) e_{ci}^{(k)}\left( v \right) dv} } \right\| }^2} - 2\int _{t - s}^t {{\rho ^\top _{ci}}\left( v \right) {\eta _{ci}}\left( v \right) dv} } \right) + \beta _{ci}^{}\int _{t - s}^t {\left( {\rho _{ci}}\left( v \right) e_{ci}^{(k)}\left( v \right) \right) ^\top dv} \underline{W_1} \end{aligned}$$

where $${\eta _{ci}} = {{e^{ - \alpha T}}{\phi _{ci}}\left( {{z_{ci}}\left( {v + T} \right) } \right) - {\phi _{ci}}\left( {{z_{ci}}\left( v \right) } \right) }$$, and $$\underline{W_1} = \underline{1}\otimes {W_1}\in R^{h_{vi}}$$.

The gradient of ${L_{i,2}}\left( k \right)$ is given as

34 $$\begin{aligned} \nabla {L_{i,2}}\left( k \right) =&\; {\left( {{\tilde{W}}_{ai}^{(k + 1)}} \right) ^\top }{\tilde{W}}_{ai}^{(k + 1)} - {\left( {{\tilde{W}}_{ai}^{(k)}} \right) ^\top }{\tilde{W}}_{ai}^{(k)} \nonumber \\ =&\; - 2{\beta _{ai}}\int _{t - s}^t { \left( {\rho _{ai}}\left( v \right) {\hat{W}}_{ai}^{(k)}\left( v \right) \right) ^\top dv} \left( {{\hat{W}}_{ai}^{(k)} - {W_{ai}^{(k)}} + {W_{ai}^{(k)}} - W_{ai}^ * } \right) + \beta _{ai}^2{\left\| {\int _{t - s}^t {{\rho _{ai}}\left( v \right) {\hat{W}}_{ai}^{(k)}\left( v \right) dv} } \right\| ^2} \end{aligned}$$

where ${W_{ai}^{(k)}}$ makes following equation hold

35 $$\begin{aligned} e_{ci}^{(k)}\left( t \right) - {e^{ - \alpha T}}{\hat{V}}_i^{(k)}\left( {t + T} \right) =&\;\int _t^{t + T} {{e^{ - \alpha \left( {v - t} \right) }}\left( {{r_1}\left( {{\delta _i}\left( v \right) } \right) + \sum \limits _{j \in {\mathscr {N}_i}} {{{\left( {u_j^{(k)}} \right) }^\top }{R_{ij}}u_j^{(k)}} } \right) } dv \nonumber \\&+ \int _t^{t + T} {{e^{ - \alpha \left( {v - t} \right) }}{{\left( {u_i^{(k)}} \right) }^\top }{R_{ii}}\psi _{ai}^\top \left( {{z_{ai}}\left( v \right) } \right) } dv W_{ai}^{(k)} \end{aligned}$$

Since ${\hat{u}}_i^{(k)}$ is bounded, there exists a scalar ${W_2}> 0$ which satisfies $\left\| {W_{ai}^{(k)} - W_{ai}^ * } \right\| ^2 \leqslant {W_2}$.

Combining Eqs. (22) and (35), one has

36 $$\begin{aligned} {\hat{V}}_i^{(k)}\left( t \right) = \int _t^{t + T} {{e^{ - \alpha \left( {v - t} \right) }}{{\left( {u_i^{(k)}} \right) }^\top }{R_{ii}}\psi _{ai}^\top \left( {{z_{ai}}\left( v \right) } \right) dv} \left( {{\hat{W}}_{ai}^{(k)} - W_{ai}^{(k)}} \right) \end{aligned}$$

Substituting Eq. (36) into Eq. (34), one has

37 $$\begin{aligned} \nabla {L_{i,2}}\left( k \right) =&\; {\beta _{ai}}\left( {{\beta _{ai}}{{\left\| {\int _{t - s}^t {{\rho _{ai}}\left( v \right) e_{ai}^{(k)}\left( v \right) dv} } \right\| }^2} - 2\int _{t - s}^t {{\rho _{ai}^\top }\left( v \right) {\eta _{ai}}\left( v \right) dv} } \right) \nonumber \\&+ \beta _{ai}^{}\int _{t - s}^t {\left( {\rho _{ai}}\left( v \right) e_{ai}^{(k)}\left( v \right) \right) ^\top dv} \left( {W_{ai}^ * - W_{ai}^{(k)}} \right) \nonumber \\ \leqslant&\; {\beta _{ai}}\left( {{\beta _{ai}}{{\left\| {\int _{t - s}^t {{\rho _{ai}}\left( v \right) e_{ai}^{(k)}\left( v \right) dv} } \right\| }^2} - 2\int _{t - s}^t {{\rho _{ai}^\top }\left( v \right) {\eta _{ai}}\left( v \right) dv} } \right) + \beta _{ai}^{}\int _{t - s}^t {\left( {\rho _{ai}}\left( v \right) e_{ai}^{(k)}\left( v \right) \right) ^\top dv} \underline{W_2} \end{aligned}$$

where $${\eta _{ai}}\left( v \right) = \int _t^{t + T} {{e^{ - \alpha \left( {v - t} \right) }}{{\left( {u_i^{(k)}} \right) }}{R_{ii}}\psi _{ai}\left( {{z_{ai}}\left( v \right) } \right) } dv$$, and $$\underline{W_2} = \underline{1}\otimes {W_2}\in R^{h_{di}}$$.

In addition, the gradient of ${L_{i,3}}\left( k \right)$ is derived as follows:

38 $$\begin{aligned} \nabla {L_{i,3}}\left( k \right) =&\; 2\delta _i^\top \delta _i+2\Theta _i \dot{V}_i(\delta )\nonumber \\ =&\; 2\delta _i^\top {\mathscr {L}_i}\left( f_e(x)+g(x)u \right) -2\Theta _i e^{-\alpha T}\left( \delta _i^ \top {Q_{ii}}{\delta _i} + u_i^ \top {R_{ii}}{u_i} + \sum \limits _{j \in {\mathscr {N}_i},i} {u_j^ \top {R_{ij}}{u_j}} \right) \nonumber \\ =&\;2\delta _i^\top {\mathscr {L}_i}f_e(x) + 2\delta _i^\top {\mathscr {L}_i}\left( \left( {{d_i} + {b_i}} \right) g\left( {{x_i}} \right) {u_i} - \sum \limits _{j \in {\mathscr {N}_i}} {{a_{ij}}g\left( {{x_j}} \right) {u_j}} \right) - 2 \Theta _i e^{-\alpha T}\left( \delta _i^ \top {Q_{ii}}{\delta _i}+\sum \limits _{j \in \{\mathscr {N}_i,i\}} {u_j^ \top {R_{ij}}{u_j}} \right) \nonumber \\ \le&\; ||{\mathscr {L}_i}f_e(x)||^2 + ||{\mathscr {L}_i}\left( d_i+b_i-\sum \limits _{j \in {\mathscr {N}_i}}a_{ij} \right) \bar{g}_i ||^2-2\Theta _i \lambda _{\text {min}}(Q_{ii}) ||\delta _i||^2 - 2\Theta _i\lambda _{\text {min}}(R_{ij})||u_i||^2 \end{aligned}$$

If $$\Theta _i$$ satisfies

$$\Theta _i> \text {max}\left\{ \frac{||{\mathscr {L}_i}f_e(x)||^2}{2\lambda _{\text {min}}(Q_{ii})}, \frac{\left\| {\mathscr {L}_i}\left( d_i+b_i-\sum \limits _{j \in {\mathscr {N}_i}}a_{ij} \right) \bar{g}_i \right\| ^2}{2\lambda _{\text {min}}(R_{ij})} \right\}$$

and the inequality $||\delta _i||^2> \frac{||{\mathscr {L}_i}f_e(x)||^2}{2\Theta _i\lambda _{\text {min}}(Q_{ii})}$ holds, one has $\nabla {L_{i,3}}\left( k \right) <0$.

Based on Eqs. (33), (37), and (38), one has

39 $$\begin{aligned} \nabla {L_i}\left( k \right) <&\; {\left\| {\int _{t - s}^t {\beta _i^{\top } {\rho _i ^{}}\left( v \right) \lambda _i\left( v \right) dv} } \right\| ^2} - 2 \beta _i^\top \int _{t - s}^t {{\rho _i}\left( v \right) \eta _i \left( v \right) dv} + \beta _i^\top \int _{t - s}^t {{\rho _i}\left( v \right) \lambda _i \left( v \right) dv} \underline{W} \end{aligned}$$

where $${\beta _i} = {\left[ {\beta _{ci}^\top \hspace{5.0pt}\beta _{ai}^\top } \right] ^\top }$$, $$\rho _i \left( v \right) = diag \{ {\rho _{ci}^\top \left( v \right) , \rho _{ai}^\top \left( v \right) } \}$$, $$\lambda _i \left( v \right) = [ ( e_{ci}^{(k)}\left( v \right) )^\top$$ $$( e_{ai}^{(k)}( v ) )^\top ]^\top$$, $$\eta _i \left( v \right) = {\left[ {\eta _{ci}^\top \left( v \right) \hspace{5.0pt}\eta _{ai}^\top \left( v \right) } \right] ^\top }$$, $$W = \max \left\{ {{W_1},{W_2}} \right\}$$, and $$\underline{W} = \underline{1} \otimes {W}\in R^{h_{vi+di}}$$.

It’s clear that $\nabla {L_i}\left( k \right) < 0$ if Eq. (26) holds, it is obtained that ${\tilde{W}}_{ci}^{(k)}$, ${\tilde{W}}_{ai}^{(k)}$, and $\delta _i$ are UUB, that is, Algorithm 2 can approximate the optimal solution instead of finding the exact optimal solution. $\square$

#### Remark 3

According to the Lyapunov-based convergence analysis^25–27^, ${\tilde{W}}_{ci}^{(k)}$, ${\tilde{W}}_{ai}^{(k)}$, and $\delta _i$ are UUB if the gradient of $L_i(k)$ can be proved to be negative under certain conditions.

#### Remark 4

It is worthwhile to note that the selections of $Q_{ii}$ and $R_{ij}$ affect the size of the ultimate bounded region $\frac{||{\mathscr {L}_i}f_e(x)||^2}{2\Theta _i\lambda _{\text {min}}(Q_{ii})}$. To minimize this region, it is recommended to select larger $Q_{ii}$ and smaller $R_{ij}$.

#### Remark 5

It should be pointed out that this study is focused on the issues of how to solve the HJB equation for nonlinear MASs, how to design an IRL controller, and how to avoid the designed IRL method from getting trapped in local optima. Meanwhile, it is noteworthy that the designed IRL method can be further optimized and extended to address more interesting issues^44–46^, such as limited communication resources, unknown disturbances, and constrained actuators.

## Simulation studies

**Fig. 1:**
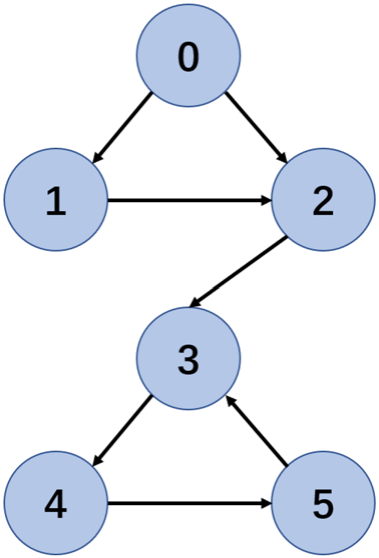
The communication topology of MASs.

In this section, two simulation examples are given to demonstrate the effectiveness of the designed method. The simulation platform is PyCharm with Python, where the sampling period, *T*, is set to 0.001 seconds. The controlled MASs consist of five agents, which are connected as shown in Fig. 1.

### Example I

In this part, a nonlinear MASs^47^ is considered, where the communication topology of the MASs is set as shown in Fig. 1. $f\left( {{x_i}} \right)$ and $g\left( {{x_i}} \right)$ of each agent are given as $$f\left( {{x_i}} \right) = \left[ \begin{aligned} {x_{i2}} \\ 0.2{x_{i2}} - 5{x_{i1}} \\ \end{aligned} \right]$$ and $$\left( {{x_i}} \right) = \left[ \begin{aligned} 0 \\ 5\cos {\left( {{x_{i2}}{x_{i1}}} \right) ^3} \\ \end{aligned} \right]$$.

The system dynamics of the leader node is $k\left( {{x_0}} \right) = \left[ {x_{02}}, - 5{x_{01}} \right] ^T$. Set ${Q_{11}} = {Q_{22}} = {Q_{33}} = {Q_{44}} = {Q_{55}} = 5$, ${R_{11}} = {R_{22}} = {R_{33}} = {R_{44}} = {R_{55}} = 0.5$, ${R_{ij}} = 0.2$ (note that ${R_{ij}} = 0.2$ only when $j \in {\mathscr {N}_i}$, or ${R_{ij}} = 0$), and the discount factor $\alpha = 0.1$. Select the sigmoid functions as the activation functions ${\phi _{ci}}\left( \cdot \right)$ and ${\psi _{ai}}\left( \cdot \right)$ of critic network and actor network, respectively. Set ${h_{vi}} = 15$, ${h_{di}} = 18$, and $s=5$. Set the learning rates ${\beta _{ci}} = 0.001$, and $\beta _{ai} = 0.0005$. Set ${N_{c,\max }} = 150$ and ${N_{a,\max }} = 100$. The thresholds are set as ${E_{c,thr}} = {E_{a,thr}} = 0.001$, and the computation error is set as $\varepsilon = 0.01$. The initial states of the leader and followers are selected as around (0, 0.5).

**Fig. 2:**
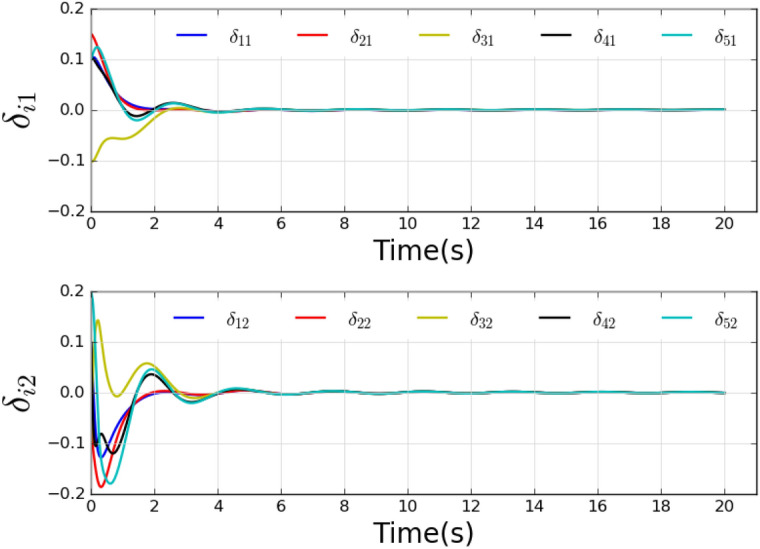
The local neighborhood tracking errors of five agents.

**Fig. 3:**
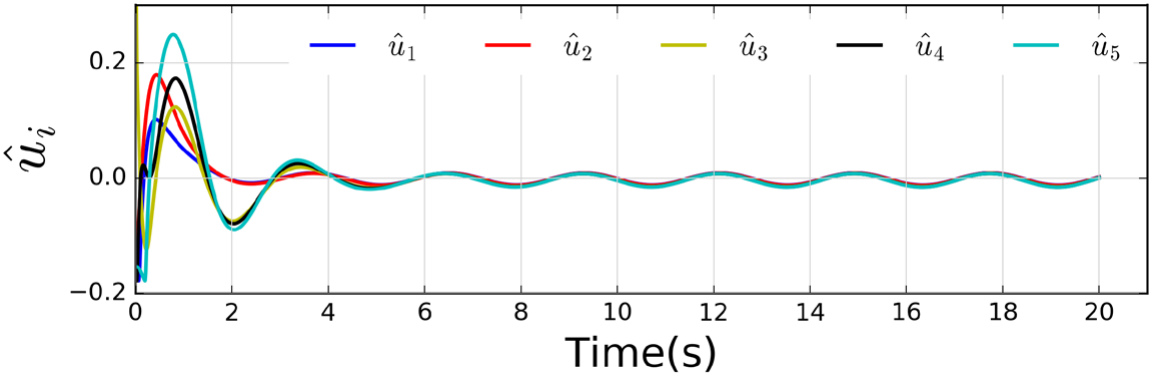
Evolutions of the control input of five agents.

**Fig. 4:**
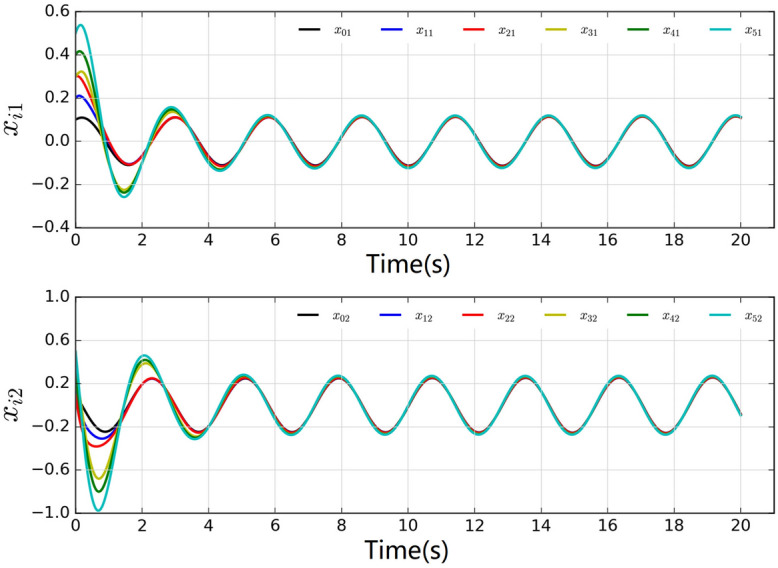
States of five agents.

The local neighborhood tracking errors are shown in Fig. 2, where it can be observed that all errors converged at about 6 seconds. Fig. 3 shows the control inputs of all agents. The states are shown in Fig. 4, which shows that all agents reach a consensus on the leader within 6 seconds.

**Fig. 5:**
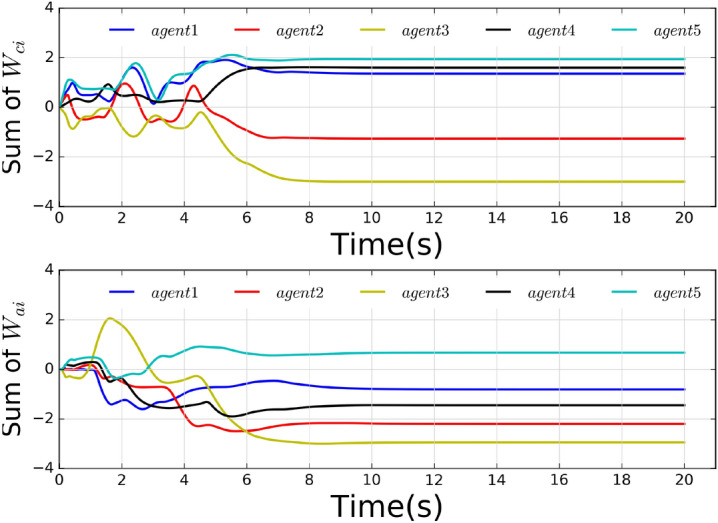
Weights of actor-critic framework.

**Fig. 6:**
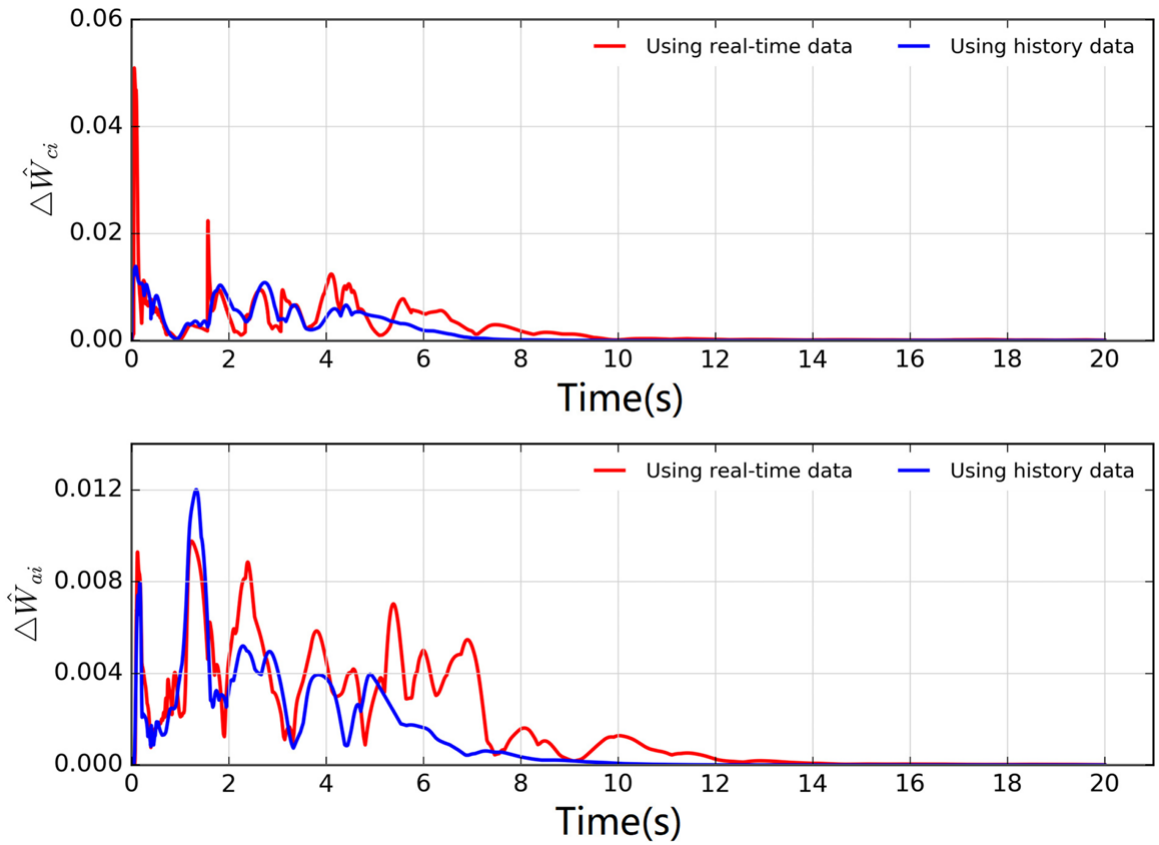
Weights’ change trend of different update rules.

Fig. 5 shows the weight curves of each agent’s critic and actor neutral networks, demonstrating that the weights are convergent. To illustrate the merits of the proposed weight update rule, we compare it with the existing method^28^. The variables $\bigtriangleup \hat{W}_{ci}(t) = {\textstyle \sum _{i\in N}^{}} || \hat{W}_{ci}(t+T) - \hat{W}_{ci}(t) ||$ and $\bigtriangleup \hat{W}_{ai}(t) = {\textstyle \sum _{i\in N}^{}}|| \hat{W}_{ai}(t+T) - \hat{W}_{ai}(t) ||$ are introduced to express the total change of the weights value. The comparison results are shown in Fig. 6, where it’s apparent that the proposed weight update rule outperforms the existing method^28^.

### Example II

**Fig. 7:**
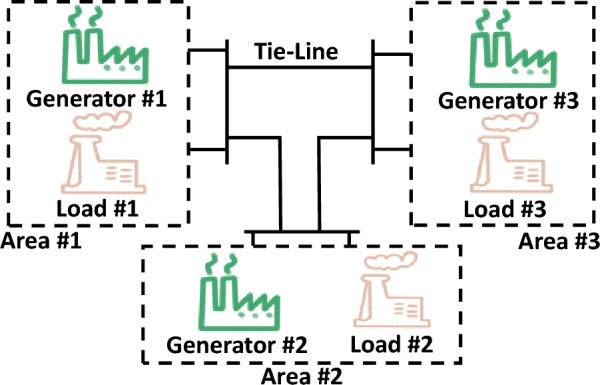
Three-area power system^49,50^.

In this section, a load frequency control simulation for a multi-area interconnected power system, shown in Fig. 7, is given to verify the effectiveness of the proposed scheme in practical systems.

The dynamics model^48^ of *i*th power system is described as

40 $$\begin{aligned} {\dot{x}_i} = f_i\left( {{x_i}} \right) + g_i\left( {{x_i}} \right) {u_i}+h_i\left( {{x_i}} \right) w_i \end{aligned}$$

where $x_i^\top = [ \Delta {F}_i, \Delta {P}_{\text{ tie-i } }, \Delta {P}_{m i}, \Delta {P}_{g i}]$ denotes the system states, $w_i^\top =[\Delta P_{d i}, \sum _{j=1, j \ne i}^N T_{i j} \Delta F_j]$ denotes the external disturbances, $$f_i(x_i) =\left[ \begin{array}{cccc} -\frac{D_i}{M_i} & -\frac{1}{M_i} & \frac{1}{M_i} & 0 \\ 2 \pi \sum _{j=1, j \ne i}^N T_{i j} & 0 & 0 & 0 \\ 0 & 0 & -\frac{1}{T_{t i}} & \frac{1}{T_{t i}} \\ -\frac{1}{R_i T_{g i}} & 0 & 0 & -\frac{1}{T_{g i}} \end{array}\right] x_i$$, $$g_i =\left[ \begin{array}{llll} 0&0&0&\frac{1}{T_{g i}} \end{array}\right] ^{\top }$$, and $$h_i =\left[ \begin{array}{llll} \beta _i&1&0&0 \end{array}\right]$$.

Moreover, $\Delta F_i$, $\Delta P_{\text{ tie }-i}$, $\Delta P_{m i}$, $\Delta P_{g i}$, and $\Delta P_{d i}$ denote the frequency deviation, the tie line power deviation, the generator output power deviation, the load change, the governor valve position deviation, and the load disturbance, respectively. The system parameters are set as $D_1 = 0.031$, $D_2 = 0.035$, $D_3 = 0.037$, $M_1 = 0.076$, $M_2 = 0.085$, $M_3 = 0.083$, $R_1 = 1.48$, $R_2 = 1.53$, $R_3 = 1.62$, $T_{g1} = 0.071$, $T_{g2} = 0.074$, $T_{g3} = 0.076$, $T_{t1} = 0.51, T_{t2} = 0.47 , T_{t3} = 0.46$, $T_{12}=T_{21}=0.22$, $T_{13}=T_{31}= 0.31$, and $T_{23}=T_{32}=0.23$. The parameters of the controller are set as ${Q_{11}} = {Q_{22}} = {Q_{33}} = {Q_{44}} = {Q_{55}} = 5$, and ${R_{11}} = {R_{22}} = {R_{33}} = {R_{44}} = {R_{55}} = 0.5$. Select the sigmoid functions as the activation functions ${\phi _{ci}}\left( \cdot \right)$ and ${\psi _{ai}}\left( \cdot \right)$ of critic neutral network and actor neural network, respectively. Set ${h_{vi}} = 6$, ${h_{di}} = 3$, and $s=10$. Set the discount factor $\alpha = 0.1$, the learning rates ${\beta _{ci}} = 0.001$, and $\beta _{ai} = 0.0005$. The weights of critic and actor neural networks are updated with ${N_{c,\max }} = 150$ and ${N_{a,\max }} = 100$, the thresholds are set as ${E_{c,thr}} = {E_{a,thr}} = 0.001$, and the computation error $\varepsilon = 0.01$. The initial states of power systems are selected as 0, and the load disturbances are $\Delta P_{di} = 0.03$ for all areas. The generation rate constraint is selected as $|\Delta \dot{P}_{c i}|\le 0.01$.

**Fig. 8:**
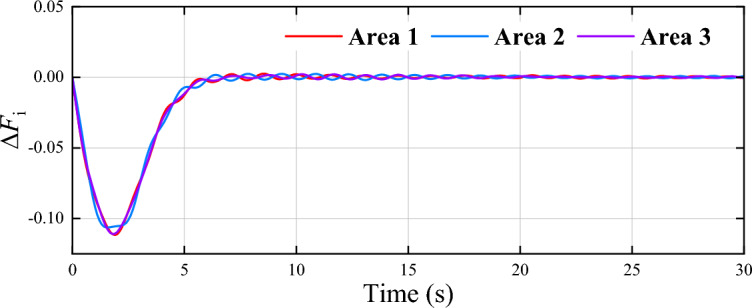
Curves of $\Delta F_i$ in three areas.

**Fig. 9:**
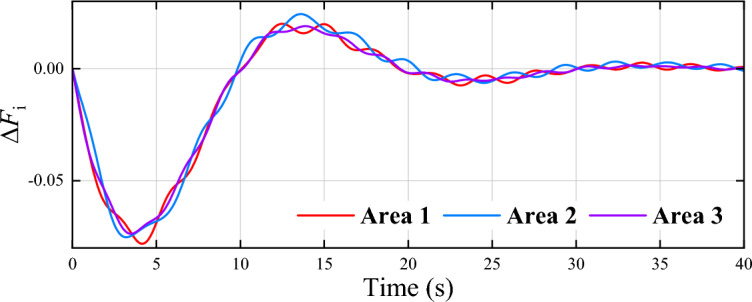
Curves of $\Delta F_i$ in three areas with the existing data-driven method^48^.

**Fig. 10:**
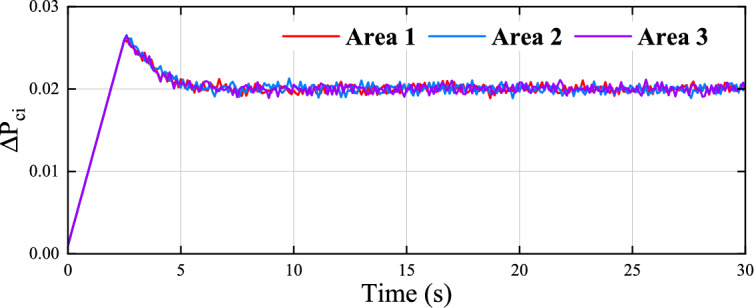
Evolution of the control input for three areas.

**Fig. 11:**
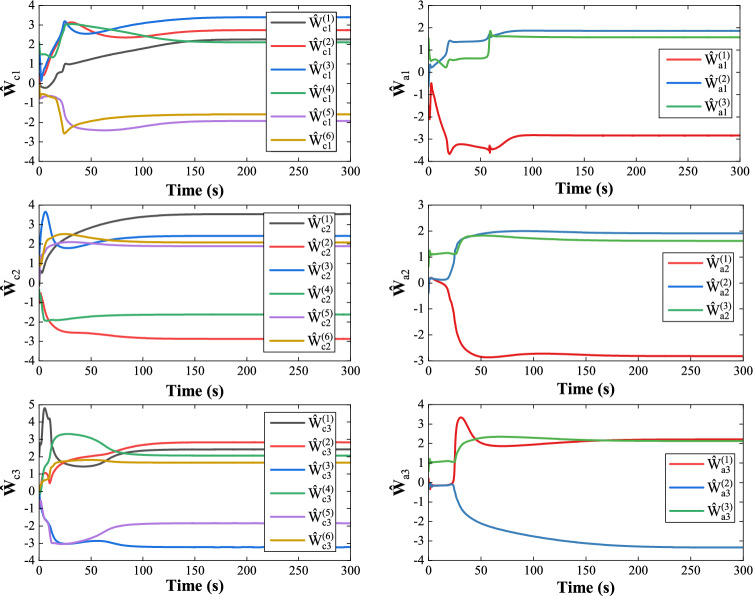
Convergence of NNs weights.

Figure 8 illustrates the frequency deviation of three areas, indicating that the proposed algorithm stabilizes the system within 7 seconds. Fig. 9 presents the control results of the existing data-driven method^48^, which exhibit higher overshoot and slower convergence compared to the proposed method. Fig. 10 shows the control input curves for the three areas, which stabilize after approximately 5 seconds. Fig. 11 illustrates the convergence of the weight parameters of the actor-critic NNs, which stabilizes around 100 seconds, indicating the completion of the training process. Collectively, Figs. 8 to 11 demonstrates that the proposed method effectively achieves load frequency control in a multi-area power system, verifying its effectiveness and applicability.

## Conclusions

This paper investigated an optimal consensus control issue for a nonlinear multi-agent system with unknown dynamics. By employing the integral reinforcement learning algorithm and policy iteration method, the control policy and value function solution have been approximated using an actor-critic neural network framework, and the historical data have been utilized in the update rule for the weights of the actor and critic neural networks. Compared to existing methods, the proposed method exhibits a faster convergence speed and effectively leverages historical information to prevent falling into local optima. Further study of limited communication resources, unknown disturbances, or constrained actuators is a meaningful endeavor in our efforts.

## Data Availability

All data generated or analysed during this study are included in this published article.
